# Association of Secondhand Smoke Exposure with Pediatric Invasive Bacterial Disease and Bacterial Carriage: A Systematic Review and Meta-analysis

**DOI:** 10.1371/journal.pmed.1000374

**Published:** 2010-12-07

**Authors:** Chien-Chang Lee, Nicole A. Middaugh, Stephen R. C. Howie, Majid Ezzati

**Affiliations:** 1 Department of Epidemiology, Harvard School of Public Health, Boston, Massachusetts, United States of America; 2 Department of Emergency Medicine, National Taiwan University Hospital, Taipei, Taiwan; 3 Bacterial Diseases Programme, Medical Research Council (UK) Laboratories, Fajara, The Gambia; 4 Department of Global Health and Population, Harvard School of Public Health, Boston, Massachusetts, United States of America; 5 Department of Environmental Health, Harvard School of Public Health, Boston, Massachusetts, United States of America; 6 Department of Epidemiology and Biostatistics, School of Public Health, Imperial College, London, United Kingdom; 7 MRC-HPA Center for Environment and Health, Imperial College, London, United Kingdom; Simon Fraser University, Canada

## Abstract

**Background:**

A number of epidemiologic studies have observed an association between secondhand smoke (SHS) exposure and pediatric invasive bacterial disease (IBD) but the evidence has not been systematically reviewed. We carried out a systematic review and meta-analysis of SHS exposure and two outcomes, IBD and pharyngeal carriage of bacteria, for *Neisseria meningitidis (N. meningitidis)*, *Haemophilus influenzae* type B (Hib), and *Streptococcus pneumoniae (S. pneumoniae*).

**Methods and Findings:**

Two independent reviewers searched Medline, EMBASE, and selected other databases, and screened articles for inclusion and exclusion criteria. We identified 30 case-control studies on SHS and IBD, and 12 cross-sectional studies on SHS and bacterial carriage. Weighted summary odd ratios (ORs) were calculated for each outcome and for studies with specific design and quality characteristics. Tests for heterogeneity and publication bias were performed. Compared with those unexposed to SHS, summary OR for SHS exposure was 2.02 (95% confidence interval [CI] 1.52–2.69) for invasive meningococcal disease, 1.21 (95% CI 0.69–2.14) for invasive pneumococcal disease, and 1.22 (95% CI 0.93–1.62) for invasive Hib disease. For pharyngeal carriage, summary OR was 1.68 (95% CI, 1.19–2.36) for *N. meningitidis*, 1.66 (95% CI 1.33–2.07) for *S. pneumoniae*, and 0.96 (95% CI 0.48–1.95) for Hib. The association between SHS exposure and invasive meningococcal and Hib diseases was consistent regardless of outcome definitions, age groups, study designs, and publication year. The effect estimates were larger in studies among children younger than 6 years of age for all three IBDs, and in studies with the more rigorous laboratory-confirmed diagnosis for invasive meningococcal disease (summary OR 3.24; 95% CI 1.72–6.13).

**Conclusions:**

When considered together with evidence from direct smoking and biological mechanisms, our systematic review and meta-analysis indicates that SHS exposure may be associated with invasive meningococcal disease. The epidemiologic evidence is currently insufficient to show an association between SHS and invasive Hib disease or pneumococcal disease. Because the burden of IBD is highest in developing countries where SHS is increasing, there is a need for high-quality studies to confirm these results, and for interventions to reduce exposure of children to SHS.

*Please see later in the article for the Editors' Summary*

## Abstract 2

Majid Ezzati and colleagues report the findings of a systematic review and meta-analysis that probes the association between environmental exposure to secondhand smoke and the epidemiology of pediatric invasive bacterial disease.

## Editors' Summary

**Background:**

The deleterious health effects of smoking on smokers are well established, but smoking also seriously damages the health of nonsmokers. Secondhand smoke (SHS), which is released by burning cigarettes and exhaled by smokers, contains hundreds of toxic chemicals that increase the risk of adults developing lung cancer and heart disease. Children, however, are particularly vulnerable to the effects of SHS exposure (also known as passive smoking) because they are still developing physically. In addition, children have little control over their indoor environment and thus can be heavily exposed to SHS. Exposure to SHS increases the risk of ear infections, asthma, respiratory symptoms (coughing, sneezing, and breathlessness), and lung infections such as pneumonia and bronchitis in young children and the risk of sudden infant death syndrome during the first year of life.

**Why Was This Study Done?:**

Several studies have also shown an association between SHS exposure (which damages the lining of the mouth, throat, and lungs and decreases immune defenses) and potentially fatal invasive bacterial disease (IBD) in children. In IBD, bacteria invade the body and grow in normally sterile sites such as the blood (bacteremia) and the covering of the brain (meningitis). Three organisms are mainly responsible for IBD in children—*Streptococcus pneumoniae*, *Haemophilus influenzae* type B (Hib), and *Neisseria meningitidis*. In 2000, *S. pneumonia* (pneumococcal disease) alone killed nearly one million children. Here, the researchers undertake a systematic review and meta-analysis of the association between SHS exposure in children and two outcomes—IBD and the presence of IBD-causing organisms in the nose and throat (bacterial carriage). A systematic review uses predefined criteria to identify all the research on a given topic; meta-analysis is a statistical method that combines the results of several studies. By combining data, it is possible to get a clearer view of the causes of a disease than is possible from individual studies.

**What Did the Researchers Do and Find?:**

The researchers identified 30 case-control studies that compared the occurrence of IBD over time in children exposed to SHS with its occurrence in children not exposed to SHS. They also identified 12 cross-sectional studies that measured bacterial carriage at a single time point in children exposed and not exposed to SHS. The researchers used the data from these studies to calculate a “summary odds ratio” (OR) for each outcome—a measure of how SHS exposure affected the likelihood of each outcome. Compared with children unexposed to SHS, exposure to SHS doubled the likelihood of invasive meningococcal disease (a summary OR for SHS exposure of 2.02). Summary ORs for invasive pneumococcal disease and Hib diseases were 1.21 and 1.22, respectively. However, these small increases in the risk of developing these IBDs were not statistically significant unlike the increase in the risk of developing meningococcal disease. That is, they might have occurred by chance. For bacterial carriage, summary ORs for SHS exposure were 1.68 for *N. meningitidis*, 1.66 for *S. pneumonia* (both these ORs were statistically significant), and 0.96 for Hib (a nonsignificant decrease in risk).

**What Do These Findings Mean?:**

These findings indicate that SHS exposure is significantly associated with invasive meningococcal disease among children. However, the evidence that SHS exposure is associated with invasive pneumococcal and Hib disease is only suggestive. These findings also indicate that exposure to SHS is associated with an increased carriage of *N. meningitidis* and *S. pneumoniae*. The accuracy and generalizability of these findings is limited by the small number of studies identified, by the lack of studies from developing countries where SHS exposure is increasing and the burden of IBD is high, and by large variations between the studies in how SHS exposure was measured and IBD diagnosed. Nevertheless, they suggest that, by reducing children's exposure to SHS (by, for example, persuading parents not to smoke at home), the illness and death caused by IBDs among children could be greatly reduced. Such a reduction would be particularly welcome in developing countries where vaccination against IBDs is low.

**Additional Information:**

Please access these Web sites via the online version of this summary at http://dx.doi.org/10.1371/journal.pmed.1000374.

- The US Centers for Disease Control and Prevention provides information on secondhand smoke, on children and secondhand smoke exposure, on meningitis, and on Hib infection
- The US Environmental Protection Agency also provides information on the health effects of exposure to secondhand smoke (in English and Spanish) and a leaflet (also in English and Spanish) entitled Secondhand Tobacco Smoke and the Health of Your Family
- The US Office of the Surgeon General provides information on the health consequences of involuntary exposure to tobacco smoke
- The World Health Organization provides a range of information on the global tobacco epidemic
- The World Health Organization has information on meningococcal disease (in English only) and on Hib (in several languages)
- The US National Foundation for Infectious Diseases provides a fact sheet on pneumococcal disease

## Introduction

Invasive bacterial disease (IBD) is an important cause of child mortality in developing and developed countries [1]–[7], accounting for at least as many child deaths as HIV/AIDS and malaria combined [6]–[8]. The organisms responsible for most pediatric IBD cases are *S. pneumoniae, Haemophilus influenzae* type B (Hib), and *N. meningitidis* [1],[2],[4],[6],[7],[9]. In 2000, there were an estimated 14.5 million cases and 826,000 deaths from pneumococcal disease in children under 5 with estimated incidence ranging from 544 per 100,000 in the Americas to 1,778 per 100,000 in Africa [6]. The burden of Hib was estimated at 8.13 million cases and 371,000 deaths with estimated incidence ranging from 504 per 100,000 in Europe to 3,627 per 100,000 in Africa [7]. While there are currently no analyses of global invasive meningococcal disease burden, regional estimates of incidence range from 0.3 to 4 cases per 100,000 in North America to as high as 1,000 cases per 100,000 in the so-called “meningitis belt” in sub-Saharan Africa [9].

Secondhand smoke (SHS; also referred to as involuntary smoking, passive smoking, or environmental tobacco smoke [ETS]) has been shown to increase the risk of several adverse outcomes in children, including lower respiratory tract infections, middle ear infection, asthma, and sudden infant death syndrome [10],[11]. Since the 1980s, epidemiologic studies have also found an association between SHS exposure and IBD or bacterial carriage, including those related to *N. meningitidis*, Hib, and *S. pneumoniae*, which suggests that SHS might be an independent risk factor for IBD. Given the persistent or growing SHS exposure in developing countries, especially in Asia, where IBD poses a major health risk, it is essential to delineate the role of exposure to SHS in the epidemiology of IBD. To our knowledge, no reviews have systematically investigated the quality and consistency of epidemiological evidence on this association, which is an important gap in our understanding of the effect of SHS exposure on the burden of infectious diseases. We carried out a systematic review and quantitative assessment of the association between SHS and the risk of IBD from *N. meningitidis*, Hib, and *S. pneumoniae* in pediatric populations, aged 1 mo to 19 y (see below). We also included the effects of SHS exposure on pharyngeal carriage of these three bacteria because asymptomatic carriage is also associated with clinical disease [12]–[16].

## Methods

### Search Strategy

We carried out a systematic literature search of SHS and IBD using Medline (via PubMed), and EMBASE from 1975 through December 2009. PubMed was searched by combining two separate queries composed of medical subject heading (MeSH) and text word (tw) keywords for the exposure and outcome of interest. The first (exposure) query was searched using the following exploded headings and independent terms: “(cigarette smoke[tw] OR “tobacco smoke pollution”[MeSH] OR “cotinine”[MeSH] OR tobacco smoke[tw] OR passive smoking[tw] OR secondhand smoke[tw] OR second hand smoke[tw] OR parental smoking[tw] OR paternal smoking[tw] OR maternal smoking[tw] OR cotinine[tw]).” The second (outcome) query was searched using exploded headings and independent terms for all three bacterial forms of IBD: (“pneumococcal infections”[MeSH] OR “*Streptococcus pneumoniae*”[MeSH] OR “*Haemophilus influenzae*”[MeSH] OR “*Neisseria meningitidis*”[MeSH] OR “Sepsis”[MeSH] OR “Meningitis, Meningococcal”[MeSH] OR “Haemophilus Infections”[MeSH] OR meningococcemia[tw] OR meningococcal[tw] OR pneumococcal[tw] OR *Haemophilus influenzae*[tw] OR h influenzae[tw] OR h influenza[tw] OR *Neisseria meningitidis*[tw]).”

We used manual restriction by age and study type (versus using automated methods in PubMed) to avoid unnecessarily eliminating any articles relevant to the search. A similar search strategy and search terms were used in EMBASE. The searches and studies included were not limited by publication date, country, or language. PubMed and EMBASE searches were conducted independently by two authors (C-CL and NAM). To ensure comprehensive acquisition of literature, independent supplemental manual searches were performed on the reference lists of relevant articles and other minor databases, including Web of Science, Cochrane databases, Centers for Disease Control and Prevention Smoking and Health Database, China National Knowledge Infrastructures (CNKI), Latin American and Caribbean of Health Sciences Information System (LILACS), and African Index Medicus (AIM). Medical Subject Heading (MeSH) and EMbase TREE tool (EMTREE) were used to guide the choice of appropriate search terms in other databases.

### Inclusion and Exclusion

Two reviewers independently identified articles eligible for in-depth examination using the following inclusion and exclusion criteria. Studies were included if at least one of the following outcomes was analyzed: invasive *S. pneumoniae* disease, invasive Hib disease, invasive *N. meningitidis* disease, and naso- or oropharyngeal carriage of any of the above three bacteria. IBD was defined as bacterial meningitis, bacterial epiglottitis, bacteremia, or microbiologically documented infection at other normally sterile sites with relevant clinical syndrome. Relevant exposures were defined as SHS or ETS exposure, parental smoking, household smoking or presence of household smoker(s), and regular contact with smokers. We excluded studies in which active smoking was the only exposure, active smoking was not distinguished from passive smoking, or studies that also included prenatal exposure. Study types included were cohort, case-control, and cross-sectional surveys, whereas case reports, review articles, editorials, and clinical guidelines were excluded. We included studies on human participants aged 1 mo to 19 y, i.e. infants, children, and adolescents. We excluded the neonatal period because of its established epidemiologic and pathophysiologic distinction from the post-neonatal period [17]. We included adolescents because age-specific *N. meningitidis* incidence peaks in childhood as well as adolescence; while *S. pneumoniae* incidence peaks in childhood and infancy, this disease may also occur in adolescents [4],[6],[9].

**Figure 1:**
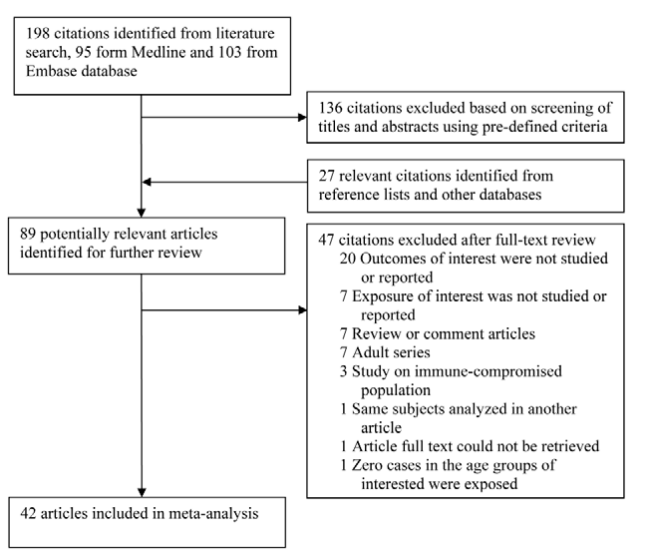
Flow chart of study identification and inclusion.

Studies on immunocompromised populations were excluded. When multiple articles reported on the same study population, we included only the most detailed publication that met the inclusion criteria. Any discrepancies on articles meriting inclusion between reviewers were resolved by a consensus meeting of three authors (C-CL, NAM, and ME). Study selection is summarized in Figure 1.

### Data Extraction and Synthesis

Data were extracted on study location, setting (e.g., community, school, hospital, etc.), population characteristics including age range and sex ratio, number of participants, definition of exposure and diagnosis of outcome, crude and adjusted effect sizes as available, and confidence intervals (CIs). We also recorded quality indicators of study design including presence of appropriate controls and covariates used for adjustment in multivariate analysis. We conducted separate analyses on IBD and bacterial carriage. When studies were identified as containing pertinent data not included in the published article (e.g., when they did not differentiate between pediatric and adult participants), we contacted the authors to obtain the missing data. When a response was not provided and raw data were provided in the article, we manually calculated the unadjusted odds ratio (OR) for inclusion in the meta-analysis. Otherwise, such articles were excluded.

### Statistical Analysis

We followed the PRISMA guidelines for meta-analysis of observational studies in our data extraction, analysis, and reporting (Text S1) [18]. Heterogeneity was tested using the Cochran Q statistic (*p<*0.05) and quantified with the *I* ^2^ statistic, which describes the variation of effect size that is attributable to heterogeneity across studies [19],[20]. The value of the *I* ^2^ statistic was used to select the appropriate pooling method: fixed-effects models were used for *I* ^2^<50% and random-effects models for *I* ^2^ ≥50% [19],[20]. CIs of *I* ^2^ were calculated by the methods suggested by Higgins et al. [21]. Pooled ORs were summarized with Mantel–Haenszel method for fixed-effect models and DerSimonian and Laird method for random effect models [20]. Galbraith plots were used to visualize the impact of individual studies on the overall homogeneity test statistic [22]. Meta-regression was used to evaluate whether effect size estimates were significantly different by specific study characteristics and quality factors, particularly those of adjustment for covariates and whether IBD diagnosis was only the more rigorous laboratory-confirmed or a mix of clinical-only and laboratory-confirmed diagnosis. We defined a study as having laboratory-confirmed diagnosis as the primary outcome if the study had more than 80% of cases confirmed by a positive culture, rapid antigen test, or PCR-based identification. In addition to meta-regression, we reestimated effects size stratified on the same study characteristics and quality factors, so that they are available as separate estimates. Even when the meta-regression result was not statistically significant, we conducted a subgroup analysis when a study characteristic was clinically or epidemiologically relevant, e.g., the age range of study participants.

The presence and the effect of publication bias was examined using a combination of the Begg and Egger tests and the “trim and fill” procedure [19],[23],[24]. This procedure considers the hypothetical possibility of studies that were missed, imputes their ORs, and recalculates a pooled OR that incorporates these hypothetical missing studies [23]. Trim-and-fill ORs are reported when the tests for publication bias were significant.

Statistical analyses were performed using Stata 10.1 (StataCorp). The metan, metabias, galbr, metareg, and metatrim macros were used for meta-analytic procedures. *p*-Values <0.05 were considered statistically significant.

## Results

### Search Results and Study Characteristics

Our search identified a total of 198 studies, of which 95 were from PubMed and 103 from EMBASE. Screening based on title and abstract identified 62 citations for full-text review (Figure 1). An additional 27 studies were identified from reference lists of the identified articles and from other databases. Of the 89 potentially relevant articles, 47 were excluded for reasons in Figure 1, leaving a total of 42 studies that met the inclusion criteria.

There were 30 studies on invasive disease and 12 on pharyngeal carriage. All invasive disease studies utilized a case-control design and all carriage studies were cross-sectional studies. The studies varied in their SHS exposure metric, age range, and case definition (Table 1). Household smoking or the presence of household smokers was the most frequent measure of SHS exposure (26 of 42 studies), while most others used maternal, paternal, or caregiver smoking. Nineteen of 30 invasive disease studies used laboratory-confirmed diagnosis as the case definition for all participants, while others had individuals with a combination of clinical-only and laboratory-confirmed diagnosis. Where clinical diagnoses were included, they were specific to the organism, including hemorrhagic rash for invasive meningococcal disease and epiglottitis for invasive Hib disease. The inclusion of carefully clinically defined cases has the potential to reduce bias in settings where pre-referral antibiotic treatment is common, either by policy or because of wide availability of antibiotics in the community. All 12 bacterial carriage studies used a culture definition, eight from nasopharyngeal swabs and the remainder from oropharyngeal swabs. The studies also varied in covariates adjusted for as shown in Table 1.

**Table 1:** Summary of studies of the association between SHS exposure and IBD or pharyngeal bacterial carriage.

Study (Location, Year)	Design	Setting and Study Population	Sample Size	Exposure^a^	Case Ascertainment^b^	Adjustment in Multivariate Analysis
**Invasive meningococcal disease**						
Haneberg et al. (Norway, 1983) [29]	Case-control	Population-based, younger than 12 y	469 (case 115)	Parental or household smoking	Laboratory-confirmed and/or clinical diagnosis	Only unadjusted ORs reported
Stuart et al. (UK, 1988) [40]	Case-control	Population-based, younger than 12 y	140 (case 70)	Household smoking	Laboratory-confirmed and/or clinical diagnosis	Only unadjusted ORs reported
Stanwell-Smith et al. (UK, 1994) [39]	Case-control	Population-based, younger than 5 y	152 (case 38)	Household smoking	Laboratory-confirmed diagnosis	Only unadjusted ORs reported
Fischer et al. (US, 1997) [27]	Case-control	Population-based, younger than 18 y	259 (case 84)	Maternal smoking at home	Laboratory-confirmed diagnosis	Age, location, maternal education, lack of primary care physician, household member density, school children density, humidifier use, church attendance
Moodley et al. (South Africa, 1999) [34]	Case-control	Population-based, younger than 14 y	280 (case 70)	Two or more household smokers	Laboratory-confirmed and/or clinical diagnosis	Age, breastfeeding, crowding index, recent respiratory tract infection, weight for age z-score
Baker et al. (New Zealand, 2000) [25]	Case-control	Population-based, younger than 8 y	515 (case 202)	Household smoking	Laboratory-confirmed and/or clinical diagnosis	Number of household members, analgesic use, attendance of social gathering, food sharing, household member respiratory tract infection symptom, recent respiratory tract infection, bed sharing
Kriz et al. (Czech Republic, 2000) [32]	Case-control	Population-based, younger than 15 y	203 (case 68)	Number of cigarettes smoked in the house in multiples of 20	Laboratory-confirmed diagnosis	Mother's and father's education, ownership of car and cottage, crowding
Hodgson A, et al. (Ghana, 2001) [30]	Case-control	Population-based, younger than 15 y	398 (case 201)	Compound (household) smoking	Laboratory-confirmed and/or clinical diagnosis	Only unadjusted ORs reported
Robinson et al. (Australia, 2001) [37]	Case-control	Population-based, younger than 16 y	141 (case 47)	Parent or partner smoking	Laboratory-confirmed and/or clinical diagnosis	Contact with dust, shared bedroom, any illness in prior 2 wk, oral muscle tone deficiency
Grein et al. (Ireland, 2001) [28]	Case-control	Population-based, younger than 6 y	354 (case 87)	Household smoking	Laboratory-confirmed diagnosis	Daycare attendance, number of children under 6 y old in household, number of adults in household, crowding index
Sorensen et al. (Denmark, 2004) [38]	Nested case-control	Nationwide population-based, younger than 18 y	9,702 (case 462)	Maternal smoking at home	ICD-8 and ICD-10 codes (treated as laboratory-confirmed and/or clinical diagnosis)	Low birth weight and prematurity, family income, crowding index
McCall et al. (Australia, 2004) [33]	Case-control	Population-based, younger than 6 y	49 (case 21)	Household smoking	Laboratory-confirmed diagnosis	Breastfeeding, room sharing, daycare attendance
Pereiro et al. (Spain, 2004) [36]	Case-control	Hospital-based, younger than 15 y	424 (case 181)	Household smoking (≥60 cigarettes/day)^c^	Laboratory-confirmed diagnosis and/or clinical diagnosis	More than four household members, meningococcal vaccination
Coen et al. (England, 2005) [26]	Case-control	Population-based, 15–19 y	288 (case 144)	Latent variable for SHS exposure based on 7 variables	Laboratory-confirmed and/or clinical diagnosis	Socioeconomic status, individual's occupation, meningococcal vaccination status
Tully et al. (UK, 2005) [41]	Case-control	Population-based, 15–19 y	228 (case 114)	Close contacts with smokers	Laboratory-confirmed and/or clinical diagnosis	Only unadjusted ORs reported
Honish et al. (Canada, 2008) [31]	Case-control	Population-based, 15–19 y	132 (case 44)	Maternal smoking	Laboratory-confirmed diagnosis	Use of humidifier, attended rave, bar visits, maternal education, visit to places where smoking was allowed, vaccination status
**Meningococcal carriage**						
Stuart et al. (UK, 1989) [58]	Cross-sectional survey	Population-based, 5–19 y	224 (case 112)	Household smoking	Nasopharyngeal carriage	Only unadjusted ORs reported
Kremastinou et al. (Greece, 1994) [55]	Cross-sectional survey	Population-based, 5–19 y	742 (case 44)	Maternal or other caretaker smoking	Oropharyngeal carriage, saliva	Only unadjusted ORs reported
Davies AL, et al. (UK,1996) [54]	Cross-sectional survey	School contacts of index case, 11–18 y	114 (case 18)	Household smoking	Nasopharyngeal carriage	Only unadjusted ORs reported
Kremastinou et al. (Greece, 1999) [56]	Cross-sectional survey	Russian immigrants, 6–15 y	625 (case 82)	Parental smoking	Oropharyngeal carriage	Only unadjusted ORs reported
MacLennan et al. (UK, 2006) [57]	Cross-sectional survey	Population-based, 15–19 y	13,919 (case 2,319)	Household smoking	Oropharyngeal carriage	Age, active smoking, intimate kissing, pub attendance, number of people in the bedroom, household member density, recent antibiotic use, school type, school size, study sites
**Invasive pneumococcal disease**						
Takala et al. (Finland, 1995) [43]	Case-control	Population-based, <15 y	433 (case 149)	Parental smoking at home	Laboratory-confirmed diagnosis	Only unadjusted ORs reported
O'Dempsey et al. (Gambia, 1996) [35]	Case-control	Population-based, 4–14.2 mo	239 (case 80)	Paternal or other household smoking	Laboratory-confirmed and/or clinical diagnosis	Mother has income, cooking smoke exposure, weight for age z-score, illness in past month, significant illness in past 6 mo
Pereiro et al. (Spain, 2004) [36]	Case-control	Hospital-based, younger than 15 y	306 (case 63)	Household smoking (≥60 cigarettes/day)^c^	Laboratory-confirmed diagnosis	Only unadjusted ORs reported
Haddad et al. (USA, 2008) [42]	Case-control	Population-based, younger than 59 mo	276 (case 120)	Tobacco exposure (no specific definition provided)	Laboratory-confirmed diagnosis	Only unadjusted ORs reported
**Pneumococcal carriage**						
Sung et al. (Hong Kong, 1995) [63]	Cross-sectional survey	Population-based, 2 mo to 5 y	921 (case 234)	Household smoking	Nasopharyngeal carriage	Only unadjusted ORs reported
Coles et al. (India, 2001) [60]	Cross-sectional survey	South India endemic area population, 6 mo	464 (case 400)	20 or more cigarettes per day were smoked in the household	Nasopharyngeal carriage	Sex, fed with colostrums, history of night blindness during pregnancy, fuel used for cooking, season, maternal education, transportation with bicycle, number of siblings younger than 5 y, vitamin A supplementation
Greenberg et al. (Israel, 2006) [61]	Cross-sectional survey	Population-based, 1–59 mo	208 (case 143)	Parental smoking at home	Nasopharyngeal carriage	Only unadjusted ORs reported
Cardozo et al. (Brazil, 2008) [59]	Cross-sectional survey	Population-based, 10–19 y	1,013 (case 83)	Household smoking	Nasopharyngeal carriage	Age, sex, presence of siblings younger than 5 y of age, upper respiratory tract infection, acute asthma
Labout et al. (Netherlands, 2008) [62]	Cross-sectional^d^	Population-based, 1.5 mo	757 (case 337)	Maternal smoking at home	Nasopharyngeal carriage	Birth weight, gestational age, parity, sex, maternal education, having siblings
**Invasive Hib disease**						
Cochi et al. (USA, 1986) [45]	Case-control	Population-based, younger than 5 y	619 (case 89)	Parental smoking	Laboratory-confirmed and/or clinical diagnosis	Only unadjusted ORs reported
Harrison et al. (USA, 1989) [3]	Case-control	Population-based, younger than 5 y	201(Case 74)	Parental smoking	Laboratory-confirmed diagnosis	Sex, race, income, age, maternal education, number of rooms in house, breastfeeding
Takala et al. (Finland, 1989) [46]	Case-control	Population-based, 1 month to 6 y	342 (case 117)	Parental smoking	Laboratory-confirmed diagnosis	Only unadjusted ORs reported
Vadheim et al. (USA,1992) [47]	Case-control	Population-based, 18–60 mo	300 (case 79)	Two or more household smokers	Laboratory-confirmed diagnosis	Age, month of diagnosis, Hib vaccine status, household size, annual household income, number of children under 5 y old, household density breastfeeding, ethnicity, daycare attendance
Arnold et al. (USA,1993) [44]	Case-control	Population-based, younger than 6 y	885 (case 295)	Household smoking	Laboratory-confirmed diagnosis	Daycare attendance, crowding index, number of children under 5 y old, maternal education, annual household income, race
Fogarty et al. (Ireland, 1995) [48]	Case-control	Population-based, younger than 14 y	435 (case 149)	Household smoking	Laboratory-confirmed diagnosis	Social class, bedroom sharing, presence of chronic illness, daycare attendance, youngest in birth order, presence of school age sibling
Muhlemann et al. (Switzerland, 1996) [51]	Case-control	Population-based, 2–16 y	438 (case 102)	Number of household smoker	Laboratory-confirmed and/or clinical diagnosis	Hib vaccination, daycare/kindergarten/school attendance, number of adult household members and siblings, siblings daycare/school attendance, bedroom sharing
Silfverdal et al. (Sweden, 1997) [52]	Case-control	Population-based, <6 y	193 (case 54)	Household smoking	Laboratory-confirmed diagnosis	Socioeconomic status, number of siblings, breastfeeding, daycare attendance, history of chronic allergy or recurrent infection
Wolff et al. (USA, 1999) [53]	Case-control	Population-based, <2 y	176 (case 60)	Parental or caretaker smoking	Laboratory-confirmed diagnosis	Only unadjusted ORs reported
Jafari et al. (USA, 1999) [49]	Case-control	Population-based, multistate, 2–16 mo	133 (case 40)	At least one household smoker	Laboratory-confirmed diagnosis	Only unadjusted ORs reported
Pereiro et al. (Spain, 2004) [36]	Case-control	Hospital-based, <5 y	154 (case 31)	Number of household smokers	Laboratory-confirmed diagnosis	More than four household members, Hib vaccination
McVernon et al. (UK, 2008) [50]	Case-control	Population based, <7 y	428 (case 138)	Household smoking	Laboratory-confirmed diagnosis	Prematurity, breastfeeding, antibiotic use, number of children in nursery, number of siblings, bedroom sharing, single parent household, central heating, home ownership, Hib vaccination
**Hib carriage**						
Ayyildiz et al. (Turkey 2003) [64]	Cross-sectional survey	Population based, 7–12 y	300 (case 9)	Household smoking	Nasopharyngeal carriage	Only unadjusted ORs reported
Oguzkaya-Artan et al. (Turkey, 2007) [65]	Cross-sectional survey	Population based, 5–7 y	683 (case 29)	Household smoking	Oropharyngeal or lower nasopharyngeal carriage	Sex, recent upper respiratory tract infection, number in the household, kindergarten attendance

a Individual studies have used different phrasing of exposure metrics. We have used “household smoking” in all cases where the definition was equivalent to smoking by household members or smoking at home.

b We defined a study as having laboratory-confirmed diagnosis if the study had more than 80% of cases confirmed by a positive culture, rapid antigen test, or PCR-based identification. Other studies were classified as a mix of laboratory-confirmed and clinical diagnosis.

c The reference category was children with no SHS exposure and the exposed group was divided into multiple categories. The OR for the highest category was used in the meta-analysis. See Results for further information about dose-response.

d Participants were enrolled in a prospective cohort study but the data for this analysis were from a cross-sectional swab, i.e., no time-to-event analysis.

ICD, International Classification of Diseases.

### SHS Exposure and IBD

#### Invasive meningococcal disease

**Figure 2:**
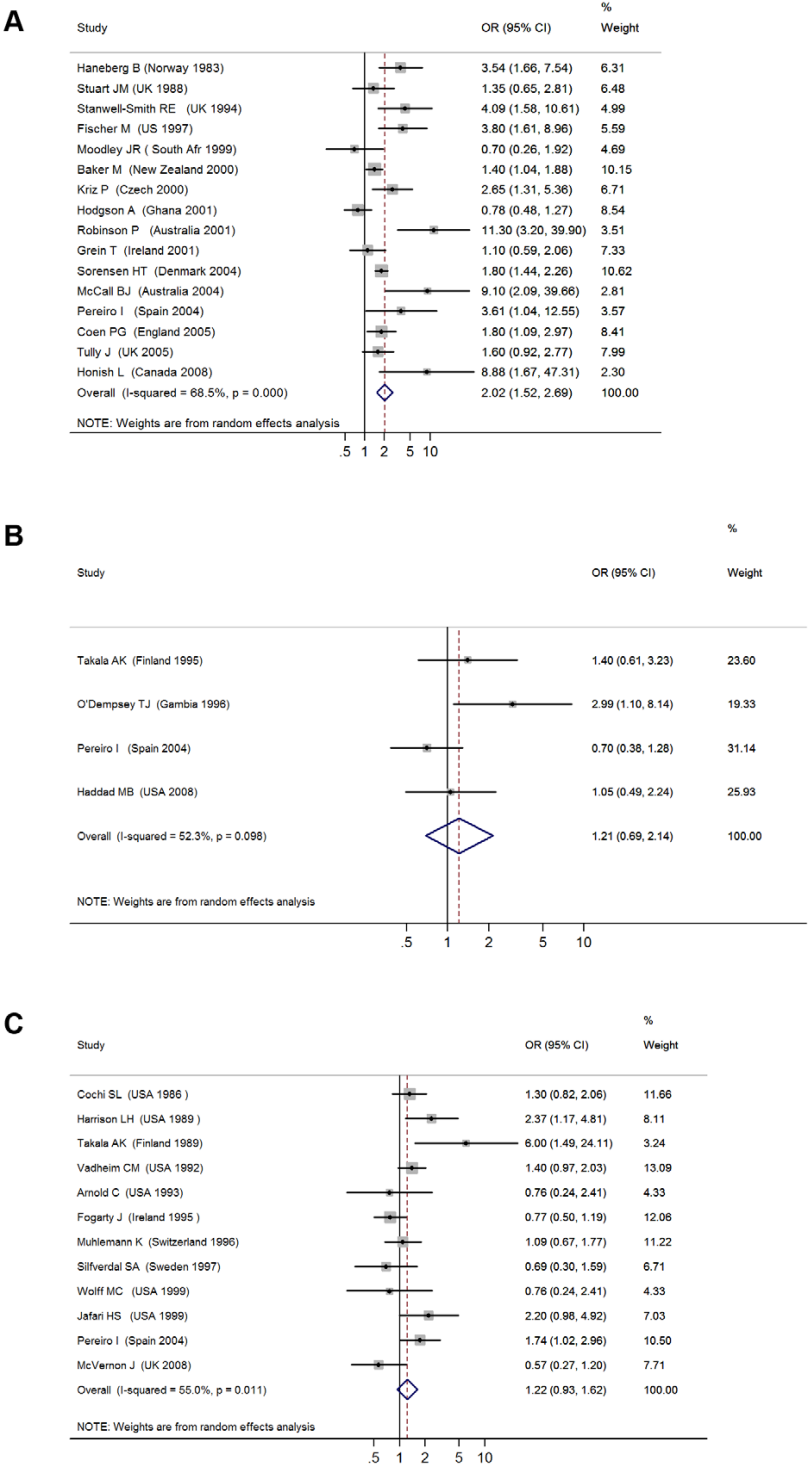
ORs for invasive bacterial disease for exposure to secondhand smoke compared to nonexposure: (A) meningococcal disease, (B) pneumococcal disease, (C) Hib disease.

The 16 studies with invasive meningococcal disease as the primary outcome included a total of 1,948 cases and 13,734 controls (Table 1) [25]–[41]. When the results from all studies were combined, SHS exposure was associated with an increased risk of invasive meningococcal disease (pooled OR 2.02, 95% CI 1.52–2.69; test of heterogeneity *p<*0.001, *I* ^2^ = 68.5%) (Figure 2A). Galbraith plots showed that two studies from Australia and Ghana were potential sources of heterogeneity [30],[37]. The effect estimate excluding these two studies was slightly reduced compared with the overall effect estimate (OR 1.79, 95% CI 1.56–2.05).

Meta-regression analysis did not show any significant effect size modification by the specific study characteristics considered, possibly because of a relatively small number of studies (Table 2). In subgroup analyses, the association was larger in studies that used laboratory-confirmed cases (OR 3.24, 95% CI 1.72–6.13) [27],[28],[31]–[33],[39]. Subgroup analysis of studies with different covariate adjustment generally found similar magnitude and direction of ORs compared with the overall effect size (Table 2) [25]–[28],[30]–[34],[36]–[40]. When the analysis was restricted to the three studies on preschool children (age ≤6 y), the association was stronger but nonsignificant (OR 3.04, 95% CI 0.89–10.47) [28],[33],[39].

**Table 2:** Summary of subgroup analysis of studies of the association of SHS and IBD or pharyngeal bacterial carriage.

Category	Number of Studies	Summary Estimate (95% CI)	*I* ^2^ (95% CI)	Meta-regression *p*-Value
**Invasive meningococcal disease**				
All studies [25]–[34],[36]–[41],[84]	16	2.02 (1.52–2.69)	68.5% (47.2–81.2)	—
Laboratory-confirmed diagnosis [27],[28],[31]–[33],[39]	6	3.24 (1.72–6.13)	63.5% (11.7–84.9)	0.149
Adjusted analysis [25]–[28],[30]–[34],[36]–[40]	11	2.18 (1.55–3.06)	66.8% (37.3–82.4)	0.500
Unadjusted analysis [29],[30],[39]–[41]	5	1.78 (0.97–3.24)	75.4% (32.1–84.7)	0.500
Adjusted for crowding, household density or room sharing [25],[27],[28],[32],[34],[36]–[38]	8	1.96 (1.36–2.82)	67.7% (34.6–93.5)	0.858
Adjusted for vaccination status [26],[31],[36]	3	2.21 (1.41–3.46)	48.6% (0–85.0)	0.468
Adjusted for breastfeeding [33],[34]	2	2.38 (0.19–29.32)	87.4%	0.894
Age less than 6 y	3	3.04 (0.88–10.47)	79.4% (34.6–93.5)	0.591
**Meningococcal carriage**				
All studies [54]–[58]	5	1.67 (1.12–2.52)	61.6% (0–85.6)	—
Adjusted analysis [57]	1	1.17 (1.05–1.30)	—	0.061
Unadjusted analysis [54]–[56],[58]	4	2.07 (1.43–2.99)	0% (0–84.7)	0.061
**Invasive pneumococcal disease**				
All studies [35],[36],[42],[43]	4	1.21 (0.69–2.14)	52.3% (0–84.2)	—
Laboratory-confirmed diagnosis [36],[42],[43]	3	0.93 (0.62–1.41)	0% (0–89.6)	0.170
Adjusted analysis [35]	1	2.99 (1.10–.8.14)	—	0.170
Unadjusted analysis [36],[42],[43]	3	0.93 (0.62–1.41)	0% (0–89.6)	0.170
Age less than 6 y [35],[42]	2	1.68 (0.61–4.66)	62.4%	0.461
Studies prior to 2000 [35],[36],[43]	3	1.33 (0.58–3.02)	68.1% (0–90.8)	0.803
**Pneumococcal carriage**				
All studies [59]–[63]	5	1.66 (1.33–2.07)	0% (0–79.2)	—
Adjusted analysis [59],[60],[62]	3	1.48 (1.01–2.16)	26.4% (0–92.4)	0.519
Unadjusted analysis [61],[63]	2	1.76 (1.34–2.31)	0%	0.519
Age less than 6 y [60],[61],[63]	3	1.63 (1.27–2.01)	12.7% (0–86.7)	0.807
**Invasive Hib disease**				
All studies [3],[36],[44]–[53]	12	1.22 (0.93–1.62)	55.0% (13.8–76.5)	—
Laboratory-confirmed diagnosis [3],[36],[44],[46]–[50],[52],[53]	10	1.24 (0.86–1.78)	62.7% (26.2–81.2)	0.929
Adjusted analysis [3],[36],[44],[47],[48],[50]–[52]	8	1.10 (0.80–1.51)	56.5% (4.2–80.2)	0.269
Unadjusted analysis [45],[46],[49],[53]	4	1.70 (0.90–3.20)	53.0% (0–84.6)	0.269
Adjusted for crowding, household density or room sharing [36],[44],[47],[48],[50],[51]	6	1.05 (0.76–1.46)	52.3% (0–81.0)	0.241
Adjusted for vaccination status [36],[47],[50]	3	1.20 (0.70–2.04)	66.9% (0–90.45)	0.915
Adjusted for breastfeeding [3],[47],[50],[52]	4	1.13 (0.63–1.98)	69.2% (11.0–89.3)	0.714
Age less than 6 y [3],[36],[44]–[47],[49],[52],[53]	9	1.46 (1.19–1.81)	38.5% (0–71.7)	0.035
Studies prior to 1990 [3],[44]–[47]	5	1.49 (1.16–1.93)	44.9% (0–79.8)	0.168
**Hib carriage**				
All studies [64],[65]	2	0.96 (0.48–1.95)	0%	—
Unadjusted analysis[65]	1	0.89 (0.42–1.89)	—	—

#### Invasive pneumococcal disease

The four case-control studies on SHS exposure and invasive pneumococcal disease included a total of 412 cases and 842 controls (Table 1) [35],[36],[42],[43]. Combined results from all studies yielded a nonsignificant association (pooled OR 1.21, 95% CI 0.69–2.14; test of heterogeneity *p = *0.098, *I* ^2^ = 52.3%) (Figure 2B). Once again, meta-regression did not show any significant effect size modification by specific study characteristics considered (Table 2). In the case of adjustment, there was only one adjusted study that had a large but imprecise effect estimate (OR 2.99, 95% CI 1.10–8.14) [35]. In subgroup analysis, the three studies with laboratory-confirmed diagnosis had a null effect size of 0.93 (95% CI 0.62–1.41). The association was stronger in studies on preschool children, but remained nonsignificant (OR 1.68, 95% CI 0.61–4.66) (Table 2) [35],[42]. Studies before 2000, when pneumococcal vaccines (including the 7-valent pneumococcal conjugate vaccine) were not widely available, also showed a stronger but nonsignificant association (OR 1.33, 95% CI 0.58–3.02) (Table 2) [3],[44]–[47].

#### Invasive Hib disease

The 12 case-control studies on SHS exposure and invasive Hib disease included a total of 1,228 cases and 3,076 controls (Table 1) [3],[36],[44]–[53]. The overall effect size was positive but nonsignificant (pooled OR 1.22, 95% CI 0.93–1.62; test of heterogeneity *p = *0.011, *I* ^2^ = 55.0%) (Figure 2C). Excluding a study in Finland that was a potential source of heterogeneity led to a lower pooled effect size, which remained nonsignificant (OR 1.18, 95% CI 0.99–1.41) [46]. Of study characteristics assessed in meta-regression, only being among preschool children was statistically significant (Table 2). In subgroup analysis, studies with laboratory-confirmed diagnosis had a similar effect size of 1.24 (95% CI 0.86–1.78), whereas adjusted studies yielded a lower and nonsignificant effect size (OR 1.10, 95% CI 0.80–1.51) [3],[36],[44],[46]–[53]. Studies on preschool children had a significant positive association (OR 1.46, 95% CI 1.19–1.81) (Table 2) [3],[36],[44]–[47],[49],[52],[53]. Studies before 1990, when Hib vaccine was not yet commonly available, had a stronger positive association (OR 1.49, 95% CI 1.16–1.93) than the overall analysis [3],[44]–[47].

### SHS Exposure and Pharyngeal Bacterial Carriage

#### *N. meningitidis* carriage

**Figure 3:**
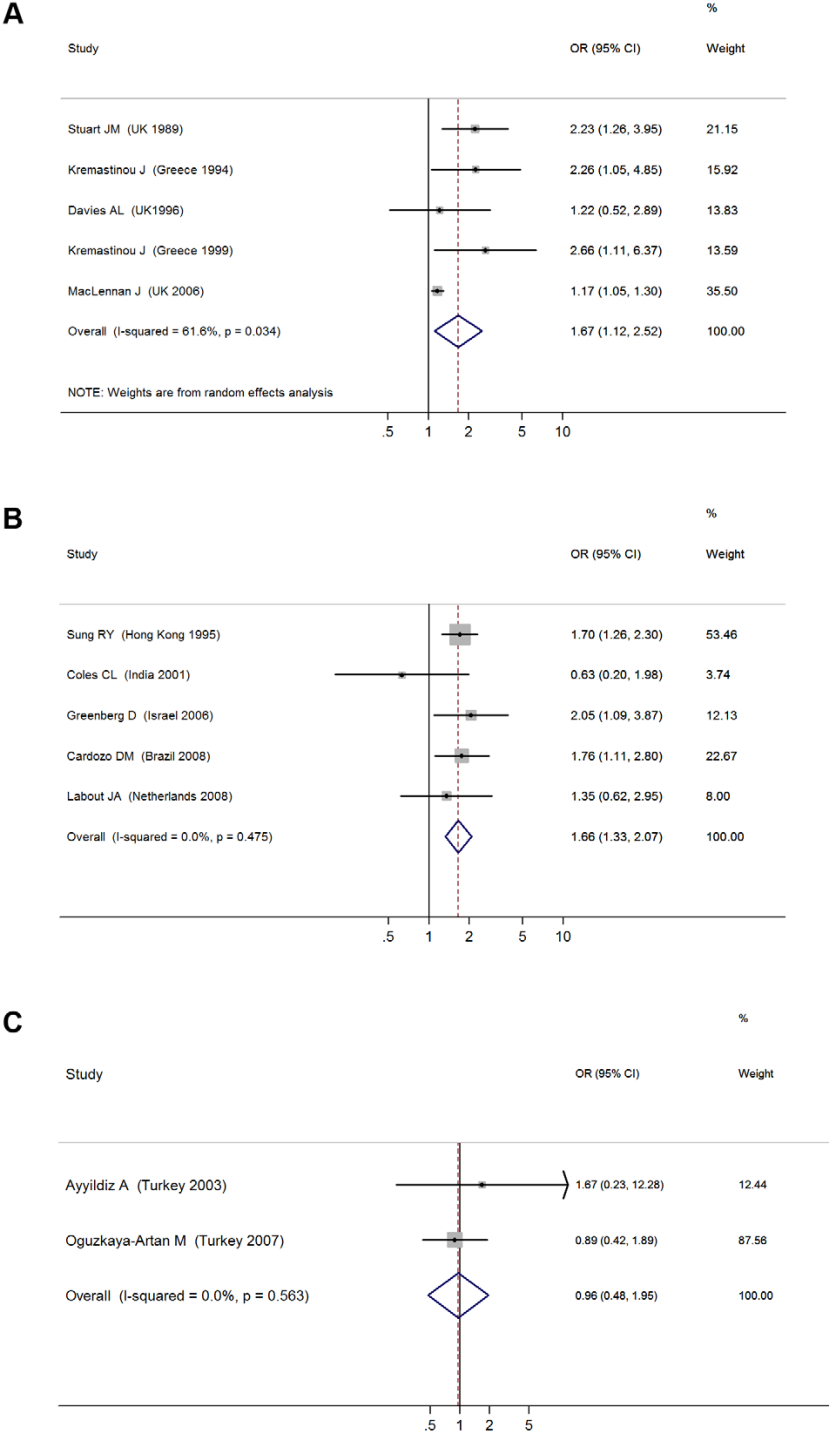
ORs for pharyngeal carriage of bacteria for exposure to secondhand smoke compared to nonexposure: (A) *N. meningitidis*, (B) *S. pneumonia*, (C) Hib.

We identified a total of five cross-sectional surveys on pharyngeal carriage of *N. meningitidis* comprising 2,575 carriers and 15,624 noncarriers (Table 1) [54]–[58]. The pooled OR for all studies showed a significant positive association between SHS exposure and pharyngeal *N. meningitidis* carriage (pooled OR 1.67, 95% CI 1.12–2.52; test of heterogeneity *p = *0.034, *I* ^2^ = 61.6%) [54]–[58] (Figure 3A). Studies with multivariate adjustment had an OR smaller than the overall analysis, but their pooled effect size remained significant (1.17 [1.05–1.30]) (Table 2) [57]. However, meta-regression analysis indicated that the difference between adjusted and crude effect sizes had borderline statistical significance (*p = *0.061).

#### *S. pneumoniae* carriage

There were five cross-sectional surveys on SHS exposure and pharyngeal carriage of *S. pneumoniae* with a total of 860 carriers and 1,746 noncarriers (Table 1) [59]–[63]. The pooled result from all studies showed a significant positive association (pooled OR 1.66, 95% CI 1.33–2.07; test of heterogeneity *p = *0.48, *I* ^2^ = 0%) (Figure 3B). Adjustment or study characteristics did not significantly modify the effect size in meta-regression analysis (Table 2). Subgroup analysis on the three studies with multivariate adjustment yielded a similar association with borderline significance (OR 1.48, 95% CI 1.01–2.16) [59],[60],[62]. Studies on preschool children also had a significant association of similar magnitude (OR 1.63, 95% CI 1.27–2.10) (Table 2) [60],[61],[63].

#### Hib carriage

There were only two cross-sectional studies on SHS exposure and childhood pharyngeal carriage of Hib that included a total of 38 cases and 945 controls (Table 1) [64],[65]. The pooled association was nonsignificant (OR 0.96, 95% CI 0.48–1.95; test of heterogeneity *p = *0.56, *I* ^2^ = 0%) (Figure 3C). The study population did not include the most vulnerable age group, i.e., preschool children.

### Dose-Response Relationships

Dose-response relationships were examined in four invasive meningococcal disease studies [28],[32],[36],[39], three invasive Hib disease studies [47],[50],[51], and one invasive pneumococcal disease study [36]. The studies had used different metrics to measure exposure and dose including number of cigarettes smoked per day and number of household smokers. The absence of a consistent definition of exposure meant that a pooled analysis of the dose-response relationship was not possible. Broadly, with the exception of the pneumococcal study and one Hib study, there was a dose-response relationship with the number of cigarettes smoked per day or the number of smokers in the household [28],[32],[36],[39],[47],[50],[51].

### Publication Bias

The test for publication bias was significant in three of the six outcomes, namely invasive meningococcal and pneumococcal diseases and *N. meningitidis* carriage (Table 3). The trim-and-fill ORs for meningococcal disease and *N. meningitidis* carriage were lower but the former remained statistically significant. The positive, but nonsignificant, OR of 1.21 (95% CI 0.69–2.14) for pneumococcal disease was replaced by a trim-and-fill OR of 0.83 (95% CI 0.45–1.53).

**Table 3:** Tests for publication bias and trim-and-fill ORs.

Outcome	Begg *p*-Value	Egger *p*-Value	Original OR (95% CI)^a^	Trim and Fill OR (95% CI)
Invasive meningococcal disease	0.015	0.060	2.02 (1.52–2.69)	1.66 (1.22–2.24)
Meningococcal carriage	0.624	0.065	1.67 (1.12–2.52)	1.32 (0.91–1.92)
Invasive pneumococcal disease	0.042	0.012	1.21 (0.69–2.14)	0.83 (0.45–1.53)
Pneumococcal carriage	0.327	0.255	1.66 (1.33–2.07)	NA
Invasive Hib disease	0.537	0.777	1.22 (0.93–1.62)	NA
Hib carriage	0.317	NA	0.96 (0.48–1.95)	NA

Trim-and-fill ORs were calculated when publication bias tests were statistically significant.

a From Figure 2.

NA, not applicable.

## Discussion

This systematic review and meta-analysis of studies on the association between SHS exposure and IBD or pharyngeal carriage of pathogenic bacteria in pediatric populations revealed a consistent and positive association between SHS exposure and invasive meningococcal disease and pharyngeal carriage of *N. meningitidis,* as well as a positive association with *S. pneumoniae* carriage. There was also a positive but not statistically significant association with invasive pneumococcal and Hib diseases. The association with Hib carriage was based on only two studies and was null. When subanalyses could be conducted, the pooled effect sizes with and without adjustment for important risk factors were generally similar, becoming slightly smaller for meningococcal and pneumococcal carriage and for invasive Hib disease, and larger for invasive meningococcal and pneumococcal diseases. Studies with laboratory-confirmed diagnosis, the more rigorous outcome, had large and statistically significant effect sizes for meningococcal disease but not for pneumococcal and Hib diseases.

The nonsignificant associations with invasive pneumococcal disease may have been partially due to the relatively small pooled sample sizes (412 cases), whereas that of Hib disease is less likely to be due to sample size (1,228 cases). For Hib disease, studies on the most vulnerable ages (≤6 y old) had larger and significant effect estimates. A factor that may have contributed to the insignificant effects may be the increasing use of Hib vaccine (since 1990) and pneumococcal conjugate vaccines (since 2000). Studies before the vaccine era had larger effect sizes for both Hib and pneumococcal diseases, but these were only statistically significant for Hib. These factors, and the strong association that has been observed between active smoking and invasive pneumococcal disease [66], should motivate additional high-quality studies with large sample sizes to clarify the role of SHS in the etiology of invasive pneumococcal disease.

There are plausible mechanisms for the effects of SHS on bacterial diseases. Both *in vivo* and *in vitro* experimental studies have found that SHS exposure may induce structural changes in the respiratory tract including peribronchiolar inflammation and fibrosis, increased mucosal permeability, and impairment of the mucociliary clearance [67],[68]. It may also decrease immune defenses, e.g., a decreased level and depressed responses of circulating immunoglobulins, decreased CD4+ lymphocyte counts and increased CD8+ lymphocyte counts, depressed phagocyte activity, and decreased release of proinflammatory cytokines [68]–[72]. All these mechanisms might increase the risk of bacterial invasion and subsequent infection. The significant findings here regarding the association of SHS exposure with bacterial carriage also support a plausible etiological role for SHS in invasive bacterial disease, because asymptomatic carriage is an intermediate step towards invasive disease [12],[13],[15],[16]. Asymptomatic carriage itself has a public health implication because it is important in population transmission of infectious bacteria[12],[15].

### Strengths and Limitations

This systematic review has strengths and limitations. To the best of our knowledge, this is the first systematic review of the epidemiologic evidence on the association between SHS exposure and pediatric IBD. We were able to include clinical invasive disease as well as the etiologically and epidemiologically important intermediate stage of asymptomatic bacterial carriage. As far as possible, we assessed sensitivity to important methodological design and quality characteristics using meta-regression and subgroup analysis. Our search covered multiple databases without language limitation.

A key limitation of our study was the relatively small number of studies, specifically from developing countries in which the IBD burden is the largest, smoking is increasing, and vaccination coverage may be lower. Notably, two African studies had a nonsignificant effect size for invasive meningococcal disease [30],[34]. One of these studies was from northern Ghana, where more than 93% of participants were exposed to fuelwood smoke [30]. In addition, negative residual confounding (due to the potential negative association of household smoking with economic status) cannot be ruled out as a source of the nonsignificant negative finding. The second study, from urban South Africa, had included an interaction term between recent upper respiratory tract infection (URTI) and SHS exposure in the multivariate analysis, which had an OR of 3.6 (95% CI 1.4–7.3) [34]. If recent URTI itself was caused by SHS and can increase the risk of IBD, then this adjustment would attenuate the true effect of the SHS term. A third African study from The Gambia had an OR of 2.99 (95% CI 1.10–8.14) for pneumococcal disease [35]. The limited number of studies from developing countries makes it difficult to assess the role of factors such as background incidence rate, nutritional status, vaccination, and coexposure to wood smoke on the ORs for SHS-IBD association. A second potential limitation is that both the exposure and outcome measurements may have been subject to error, which is likely to have biased our results towards the null and reduced its significance. Third, heterogeneity of effect sizes across studies restricts our ability and confidence to generalize the results of this pooled data analysis to all populations. Fourth, the studies on association with bacterial carriage were distinct from those on IBD and no SHS-IBD studies had assessed carriage at baseline. As a result, we were not able to assess whether SHS exposure only increases the risk for colonization, or increases the risk of subsequent infection, or both. Fifth, because IBD is a complex disease with multiple causes, there is potential for residual confounding in the observational studies included in our analysis. This is especially relevant given that the currently available SHS-IBD studies were case-control or nested case-control studies and those on SHS-bacterial carriage were cross-sectional studies. Our findings on the potential causal associations should motivate new prospective studies. Sixth, our study focused on pediatric SHS exposure and did not assess studies on perinatal SHS exposure as a risk factor for IBD, with some of the effect possibly mediated through low birth weight. [73],[74]. Finally, while we assessed the potential for publication bias and report trim-and-fill ORs, the latter estimates are themselves subject to methodologic limitations especially when the number of studies is small [75].

Despite the limitations of current epidemiologic studies, our meta-analysis provides some evidence of an association between SHS and IBD and pharyngeal carriage, especially in preschool children. Although there are efficacious vaccines against all three pathogens assessed in this study, many children in low-income countries are not covered in routine immunizations and have limited access to case management [76]–[80]. Vaccine pricing remains an obstacle to uptake, while waning immunity and serotype replacement may undermine long-term vaccine effectiveness [78],[81]–[83]. Thus scaling up vaccine coverage and case management must be accompanied by nonvaccine interventions, such as environmental interventions, to address the large burden of IBDs. Tobacco smoking and SHS exposure have increased in low-income and middle-income countries, making SHS exposure a global problem [84],[85]. While public smoking bans have been effective in reducing adult SHS exposure and adverse health effects [86],[87], children's exposure to SHS may occur at home, where bans may be difficult to enforce [11]. An estimated 700 million children worldwide are exposed to SHS at home [84]. If the observed effects are causal, our results indicate that in a population where 25% of young children are exposed to SHS (e.g., Brazil or South Africa), 5%–20% of IBD cases may be attributable to this risk factor; the attributable fraction would be 10%–34% in populations where exposure is 50% (e.g., Egypt or Indonesia). Effects of such magnitude should motivate a number of research and intervention steps specifically related to SHS and pediatric IBD: Firstly, there should be well-designed prospective studies with high-quality measurement of exposure, outcome, and potential confounders to overcome the limitations of the current studies. Secondly, interventions that specifically focus on reducing children's exposure at home, schools, and other environments should be pursued. These two directions are particularly important in developing countries where the IBD burden is high and exposure to SHS is high or increasing. Finally, the effects of other combustion pollutant sources that are common in developing countries, especially smoke from wood and animal dung fuels, on IBD should be subject to research.

## Supporting Information

Text S1 PRISMA checklist.
(0.07 MB DOC)

## Abbreviations

CI: confidence interval
Hib: *Haemophilus influenzae* type B
IBD: invasive bacterial disease
MeSH: medical subject heading
SHS: secondhand smoke
tw: text word

## References

[1] Berkley JA, Lowe BS, Mwangi I, Williams T, Bauni E (2005). Bacteremia among children admitted to a rural hospital in Kenya.. N Engl J Med.

[2] Brent AJ, Ahmed I, Ndiritu M, Lewa P, Ngetsa C (2006). Incidence of clinically significant bacteraemia in children who present to hospital in Kenya: community-based observational study.. Lancet.

[3] Harrison LH, Broome CV, Hightower AW (1989). Haemophilus influenzae type b polysaccharide vaccine: an efficacy study. Haemophilus Vaccine Efficacy Study Group.. Pediatrics.

[4] Kaijalainen T, Kharit SM, Kvetnaya AS, Sirkia K, Herva E (2008). Invasive infections caused by Neisseria meningitidis, Haemophilus influenzae and Streptococcus pneumoniae among children in St Petersburg, Russia.. Clin Microbiol Infect.

[5] Mulholland EK, Adegbola RA (2005). Bacterial infections–a major cause of death among children in Africa.. N Engl J Med.

[6] O'Brien KL, Wolfson LJ, Watt JP, Henkle E, Deloria-Knoll M (2009). Burden of disease caused by Streptococcus pneumoniae in children younger than 5 years: global estimates.. Lancet.

[7] Watt JP, Wolfson LJ, O'Brien KL, Henkle E, Deloria-Knoll M (2009). Burden of disease caused by Haemophilus influenzae type b in children younger than 5 years: global estimates.. Lancet.

[8] World Health Organization (2005). Make every mother and child count..

[9] Harrison LH, Trotter CL, Ramsay ME (2009). Global epidemiology of meningococcal disease.. Vaccine.

[10] California Environmental Protection Agency (2005). Proposed Identification of Environmental Tobacco Smoke as a TAC; Office of Environmental Health Hazard Assessment CEPA, editor..

[11] Office of the Surgeon General (2006). The health consequences of involuntary exposure to tobacco smoke: a report of the Surgeon General; Department of Health and Human Services C, editor..

[12] Bogaert D, De Groot R, Hermans PW (2004). Streptococcus pneumoniae colonization: the key to pneumococcal disease.. Lancet Infect Dis.

[13] Gray BM, Converse GM, Dillon HC (1980). Epidemiologic studies of Streptococcus pneumoniae in infants: acquisition, carriage, and infection during the first 24 months of life.. J Infect Dis.

[14] Lloyd-Evans N, O'Dempsey TJ, Baldeh I, Secka O, Demba E (1996). Nasopharyngeal carriage of pneumococci in Gambian children and in their families.. Pediatr Infect Dis J.

[15] van der Poll T, Opal SM (2009). Pathogenesis, treatment, and prevention of pneumococcal pneumonia.. Lancet.

[16] van Deuren M, Brandtzaeg P, van der Meer JW (2000). Update on meningococcal disease with emphasis on pathogenesis and clinical management.. Clin Microbiol Rev.

[17] Vergnano S, Sharland M, Kazembe P, Mwansambo C, Heath PT (2005). Neonatal sepsis: an international perspective.. Arch Dis Child Fetal Neonatal Ed.

[18] Moher D, Liberati A, Tetzlaff J, Altman DG (2009). Preferred reporting items for systematic reviews and meta-analyses: the PRISMA statement.. Ann Intern Med.

[19] Higgins JP, Thompson SG, Deeks JJ, Altman DG (2003). Measuring inconsistency in meta-analyses.. BMJ.

[20] DerSimonian R, Laird N (1986). Meta-analysis in clinical trials.. Control Clin Trials.

[21] Higgins JP, Thompson SG (2002). Quantifying heterogeneity in a meta-analysis.. Stat Med.

[22] Galbraith RF (1988). A note on graphical presentation of estimated odds ratios from several clinical trials.. Stat Med.

[23] Duval S, Tweedie R (2000). Trim and fill: a simple funnel-plot-based method of testing and adjusting for publication bias in meta-analysis.. Biometrics.

[24] Egger M, Davey Smith G, Schneider M, Minder C (1997). Bias in meta-analysis detected by a simple, graphical test.. BMJ.

[25] Baker M, McNicholas A, Garrett N, Jones N, Stewart J (2000). Household crowding a major risk factor for epidemic meningococcal disease in Auckland children.. Pediatr Infect Dis J.

[26] Coen PG, Tully J, Stuart JM, Ashby D, Viner RM (2006). Is it exposure to cigarette smoke or to smokers which increases the risk of meningococcal disease in teenagers?. Int J Epidemiol.

[27] Fischer M, Hedberg K, Cardosi P, Plikaytis BD, Hoesly FC (1997). Tobacco smoke as a risk factor for meningococcal disease.. Pediatr Infect Dis J.

[28] Grein T, O'Flanagan D (2001). Day-care and meningococcal disease in young children.. Epidemiol Infect.

[29] Haneberg B, Tonjum T, Rodahl K, Gedde-Dahl TW (1983). Factors preceding the onset of meningococcal disease, with special emphasis on passive smoking, symptoms of ill health.. NIPH Ann.

[30] Hodgson A, Smith T, Gagneux S, Adjuik M, Pluschke G (2001). Risk factors for meningococcal meningitis in northern Ghana.. Trans R Soc Trop Med Hyg.

[31] Honish L, Soskolne CL, Senthilselvan A, Houston S (2008). Modifiable risk factors for invasive meningococcal disease during an Edmonton, Alberta outbreak, 1999-2002.. Can J Public Health.

[32] Kriz P, Bobak M, Kriz B (2000). Parental smoking, socioeconomic factors, and risk of invasive meningococcal disease in children: a population based case-control study.. Arch Dis Child.

[33] McCall BJ, Neill AS, Young MM (2004). Risk factors for invasive meningococcal disease in southern Queensland, 2000-2001.. Intern Med J.

[34] Moodley JR, Coetzee N, Hussey G (1999). Risk factors for meningococcal disease in Cape Town.. S Afr Med J.

[35] O'Dempsey TJ, McArdle TF, Morris J, Lloyd-Evans N, Baldeh I (1996). A study of risk factors for pneumococcal disease among children in a rural area of west Africa.. Int J Epidemiol.

[36] Pereiro I, Diez-Domingo J, Segarra L, Ballester A, Albert A (2004). Risk factors for invasive disease among children in Spain.. J Infect.

[37] Robinson P, Taylor K, Nolan T (2001). Risk-factors for meningococcal disease in Victoria, Australia, in 1997.. Epidemiol Infect.

[38] Sørensen HT, Labouriau R, Jensen ES, Mortensen PB, Schønheyder HC (2004). Fetal growth, maternal prenatal smoking, and risk of invasive meningococcal disease: a nationwide case-control study.. Int J Epidemiol.

[39] Stanwell-Smith RE, Stuart JM, Hughes AO, Robinson P, Griffin MB (1994). Smoking, the environment and meningococcal disease: a case control study.. Epidemiol Infect.

[40] Stuart JM, Cartwright KA, Dawson JA, Rickard J, Noah ND (1988). Risk factors for meningococcal disease: a case control study in south west England.. Community Med.

[41] Tully J, Viner RM, Coen PG, Stuart JM, Zambon M (2006). Risk and protective factors for meningococcal disease in adolescents: matched cohort study.. BMJ.

[42] Haddad MB, Porucznik CA, Joyce KE, De AK, Pavia AT (2008). Risk factors for pediatric invasive pneumococcal disease in the Intermountain West, 1996-2002.. Ann Epidemiol.

[43] Takala AK, Jero J, Kela E, Ronnberg PR, Koskenniemi E (1995). Risk factors for primary invasive pneumococcal disease among children in Finland.. JAMA.

[44] Arnold C, Makintube S, Istre GR (1993). Day care attendance and other risk factors for invasive Haemophilus influenzae type b disease.. Am J Epidemiol.

[45] Cochi SL, Fleming DW, Hightower AW, Limpakarnjanarat K, Facklam RR (1986). Primary invasive Haemophilus influenzae type b disease: a population-based assessment of risk factors.. J Pediatr.

[46] Takala AK, Eskola J, Palmgren J, Ronnberg PR, Kela E (1989). Risk factors of invasive Haemophilus influenzae type b disease among children in Finland.. J Pediatr.

[47] Vadheim CM, Greenberg DP, Bordenave N, Ziontz L, Christenson P (1992). Risk factors for invasive Haemophilus influenzae type b in Los Angeles County children 18-60 months of age.. Am J Epidemiol.

[48] Fogarty J, Moloney AC, Newell JB (1995). The epidemiology of Haemophilus influenzae type b disease in the Republic of Ireland.. Epidemiol Infect.

[49] Jafari HS, Adams WG, Robinson KA, Plikaytis BD, Wenger JD (1999). Efficacy of Haemophilus influenzae type b conjugate vaccines and persistence of disease in disadvantaged populations. The Haemophilus Influenzae Study Group.. Am J Public Health.

[50] McVernon J, Andrews N, Slack M, Moxon R, Ramsay M (2008). Host and environmental factors associated with Hib in England, 1998-2002.. Arch Dis Child.

[51] Muhlemann K, Alexander ER, Weiss NS, Pepe M, Schopfer K (1996). Risk factors for invasive Haemophilus influenzae disease among children 2-16 years of age in the vaccine era, Switzerland 1991-1993. The Swiss H. Influenzae Study Group.. Int J Epidemiol.

[52] Silfverdal SA, Bodin L, Hugosson S, Garpenholt O, Werner B (1997). Protective effect of breastfeeding on invasive Haemophilus influenzae infection: a case-control study in Swedish preschool children.. Int J Epidemiol.

[53] Wolff MC, Moulton LH, Newcomer W, Reid R, Santosham M (1999). A case-control study of risk factors for Haemophilus influenzae type B disease in Navajo children.. Am J Trop Med Hyg.

[54] Davies AL, O'Flanagan D, Salmon RL, Coleman TJ (1996). Risk factors for Neisseria meningitidis carriage in a school during a community outbreak of meningococcal infection.. Epidemiol Infect.

[55] Kremastinou J, Blackwell C, Tzanakaki G, Kallergi C, Elton R (1994). Parental smoking and carriage of Neisseria meningitidis among Greek schoolchildren.. Scand J Infect Dis.

[56] Kremastinou J, Tzanakaki G, Velonakis E, Voyiatzi A, Nickolaou A (1999). Carriage of Neisseria meningitidis and Neisseria lactamica among ethnic Greek school children from Russian immigrant families in Athens.. FEMS Immunol Med Microbiol.

[57] MacLennan J, Kafatos G, Neal K, Andrews N, Cameron JC (2006). Social behavior and meningococcal carriage in British teenagers.. Emerg Infect Dis.

[58] Stuart JM, Cartwright KA, Robinson PM, Noah ND (1989). Effect of smoking on meningococcal carriage.. Lancet.

[59] Cardozo DM, Nascimento-Carvalho CM, Andrade AL, Silvany-Neto AM, Daltro CH (2008). Prevalence and risk factors for nasopharyngeal carriage of Streptococcus pneumoniae among adolescents.. J Med Microbiol.

[60] Coles CL, Kanungo R, Rahmathullah L, Thulasiraj RD, Katz J (2001). Pneumococcal nasopharyngeal colonization in young South Indian infants.. Pediatr Infect Dis J.

[61] Greenberg D, Givon-Lavi N, Broides A, Blancovich I, Peled N (2006). The contribution of smoking and exposure to tobacco smoke to Streptococcus pneumoniae and Haemophilus influenzae carriage in children and their mothers.. Clin Infect Dis.

[62] Labout JA, Duijts L, Arends LR, Jaddoe VW, Hofman A (2008). Factors associated with pneumococcal carriage in healthy Dutch infants: the generation R study.. J Pediatr.

[63] Sung RY, Ling JM, Fung SM, Oppenheimer SJ, Crook DW (1995). Carriage of Haemophilus influenzae and Streptococcus pneumoniae in healthy Chinese and Vietnamese children in Hong Kong.. Acta Paediatr.

[64] Ayyildiz A, Aktas AE, Yazgi H (2003). Nasopharyngeal carriage rate of Haemophilus influenzae in children aged 7-12 years in Turkey.. Int J Clin Pract.

[65] Oguzkaya-Artan M, Baykan Z, Artan C (2008). Nasal carriage of Staphylococcus aureus in healthy preschool children.. Jpn J Infect Dis.

[66] Nuorti JP, Butler JC, Farley MM, Harrison LH, McGeer A (2000). Cigarette smoking and invasive pneumococcal disease. Active Bacterial Core Surveillance Team.. N Engl J Med.

[67] Dye JA, Adler KB (1994). Effects of cigarette smoke on epithelial cells of the respiratory tract.. Thorax.

[68] Wanner A, Salathé M, O'Riordan TG (1996). Mucociliary clearance in the airways.. Am J Respir Crit Care Med.

[69] Arcavi L, Benowitz NL (2004). Cigarette smoking and infection.. Arch Intern Med.

[70] Gaschler GJ, Zavitz CC, Bauer CM, Skrtic M, Lindahl M (2008). Cigarette smoke exposure attenuates cytokine production by mouse alveolar macrophages.. Am J Respir Cell Mol Biol.

[71] Green RM, Gally F, Keeney JG, Alper S, Gao B (2009). Impact of cigarette smoke exposure on innate immunity: a Caenorhabditis elegans model.. PLoS One.

[72] Martí-Lliteras P, Regueiro V, Morey P, Hood DW, Saus C (2009). Nontypeable Haemophilus influenzae clearance by alveolar macrophages is impaired by exposure to cigarette smoke.. Infect Immun.

[73] Sorensen HT, Labouriau R, Jensen ES, Mortensen PB, Schonheyder HC (2004). Fetal growth, maternal prenatal smoking, and risk of invasive meningococcal disease: a nationwide case-control study.. Int J Epidemiol.

[74] Yusuf HR, Rochat RW, Baughman WS, Gargiullo PM, Perkins BA (1999). Maternal cigarette smoking and invasive meningococcal disease: a cohort study among young children in metropolitan Atlanta, 1989-1996.. Am J Public Health.

[75] Moreno SG, Sutton AJ, Turner EH, Abrams KR, Cooper NJ (2009). Novel methods to deal with publication biases: secondary analysis of antidepressant trials in the FDA trial registry database and related journal publications.. BMJ.

[76] Centers for Disease Control and Prevention (CDC) (2005). Direct and indirect effects of routine vaccination of children with 7-valent pneumococcal conjugate vaccine on incidence of invasive pneumococcal disease–United States, 1998-2003.. MMWR Morb Mortal Wkly Rep.

[77] De Wals P, Deceuninck G, Boulianne N, De Serres G (2004). Effectiveness of a mass immunization campaign using serogroup C meningococcal conjugate vaccine.. JAMA.

[78] Levine OS, O'Brien KL, Knoll M, Adegbola RA, Black S (2006). Pneumococcal vaccination in developing countries.. Lancet.

[79] Lim SS, Stein DB, Charrow A, Murray CJ (2008). Tracking progress towards universal childhood immunisation and the impact of global initiatives: a systematic analysis of three-dose diphtheria, tetanus, and pertussis immunisation coverage.. Lancet.

[80] Morris SK, Moss WJ, Halsey N (2008). Haemophilus influenzae type b conjugate vaccine use and effectiveness.. Lancet Infect Dis.

[81] Madhi SA, Adrian P, Kuwanda L, Cutland C, Albrich WC (2007). Long-term effect of pneumococcal conjugate vaccine on nasopharyngeal colonization by Streptococcus pneumoniae–and associated interactions with Staphylococcus aureus and Haemophilus influenzae colonization–in HIV-Infected and HIV-uninfected children.. J Infect Dis.

[82] Madhi SA, Adrian P, Kuwanda L, Jassat W, Jones S (2007). Long-term immunogenicity and efficacy of a 9-valent conjugate pneumococcal vaccine in human immunodeficient virus infected and non-infected children in the absence of a booster dose of vaccine.. Vaccine.

[83] Obaro SK, Adegbola RA, Banya WA, Greenwood BM (1996). Carriage of pneumococci after pneumococcal vaccination.. Lancet.

[84] World Health Organization (2009). WHO Report on the Global Tobacco Epidemic: implementing smoke-free environments..

[85] Jha P, Chaloupka FJ (2000). Tobacco control in developing countries..

[86] Lightwood JM, Glantz SA (2009). Declines in acute myocardial infarction after smoke-free laws and individual risk attributable to secondhand smoke.. Circulation.

[87] Sargent RP, Shepard RM, Glantz SA (2004). Reduced incidence of admissions for myocardial infarction associated with public smoking ban: before and after study.. BMJ.

